# Impact of preoperative antiviral therapy on the prognosis of hepatitis B virus-related hepatocellular carcinoma

**DOI:** 10.1186/s12885-024-12031-0

**Published:** 2024-03-04

**Authors:** Yuxin Liang, Deyuan Zhong, Zilong Zhang, Yuhao Su, Su Yan, Chunyou Lai, Yutong Yao, Ying Shi, Xiaolun Huang, Jin Shang

**Affiliations:** 1 Present Address: Liver Transplantation Center and HBP Surgery, Sichuan Cancer Hospital & Institute, Sichuan Cancer Center, School of Medicine, University of Electronic Science and Technology of China, Chengdu, China; 2 Department of Hepatobiliary-Pancreatic Surgery, Cell Transplantation Center, Sichuan Provincial People’s Hospital, University of Electronic Science and Technology of China, No. 32, West Second Section, First Ring Road, Qingyang District, Chengdu, 610072 China; 3 Clinical Immunology Translational Medicine Key Laboratory of Sichuan Province & Organ Transplant Research Institute, Sichuan Provincial People’s Hospital, University of Electronic Science and Technology of China, No. 32, West Second Section, First Ring Road, Qingyang District, Chengdu, 610072 China; 4 Department of Hepatobiliary-Pancreatic and Hernia Surgery, Wuhan Fourth Hospital, Puai Hospital, Tongji Medical College, Huazhong University of Science and Technology, Wuhan, 430033 China

**Keywords:** Anti-PD-1 therapy, HCC, Curative resection, Recurrence, Survival

## Abstract

**Background:**

For chronic hepatitis B virus (HBV) infection patients, increasing evidence has demonstrated the effectiveness of expanding the indications and applicable population for antiviral therapy. However, the expanded indication of antiviral therapy for hepatocellular carcinoma (HCC) remains to be further explored.

**Methods:**

196 HBV-related HCC patients who received radical hepatectomy and nucleos(t)ide analogues (NAs) therapy at Sichuan Provincial People’s Hospital were enrolled in this study. HCC recurrence, overall survival (OS), early virological (VR) and biochemical responses (BR) of patients were compared between different NAs therapy and the use of anti-programmed cell death protein 1 (PD-1) therapy.

**Results:**

NAs therapy at different timing of surgery was a strong independent risk factor for postoperative recurrence and overall mortality of HBV-related HCC patients. Furthermore, in HCC patients who received postoperative anti-PD-1 therapy, patients with HBV DNA < 1000 copy/mL had significantly better recurrence-free survival (RFS) and OS than those with HBV DNA ≥ 1000 copy/mL (HR: 7.783; P = 0.002; HR: 6.699; P < 0.001). However, the differences of RFS and OS rates between entecavir group and tenofovir disoproxil fumarate group were not statistically significant. Similar results were also observed in the rates of early VR, BR and combined VR and BR.

**Conclusion:**

Timely and reasonable preoperative NAs therapy showed clinical benefit in improving the prognosis of patients with HBV-related HCC, even in the case of normal alanine aminotransferase (ALT) level and negative hepatitis e antigen (HBeAg). Furthermore, a possible synergistic effect between antiviral therapy and anti-PD-1 therapy was founded and need further verification.

**Supplementary Information:**

The online version contains supplementary material available at 10.1186/s12885-024-12031-0.

## Introduction

As the sixth most common malignancy and the second highest cause of cancer-related mortality worldwide, hepatocellular carcinoma (HCC) is most prevalent in Asian patients due to chronic hepatitis B virus (HBV) infection [1–3]. Although curative resection remains to be the first-line treatment for HCC, the prognosis is still poor due to the high rate of postoperative intrahepatic recurrence in the residual fibrotic or cirrhotic liver [4–6]. To date, there is still no consensus on postoperative adjuvant therapy for patients with HCC, while the effectiveness of these adjunctive therapies has been demonstrated by published studies, including antiviral therapy, transarterial chemoembolization (TACE), molecularly targeted therapy, immunotherapy, etc. [7].

HBV replication has been proven to be a significant risk factor for HCC development, hepatic decompensation, and liver-related mortality [8–10]. Therefore, using antiviral therapy to reduce HBV viral load could lower the risk of HCC recurrence and improve postoperative patient survival [8, 11–13]. Interferon and nucleos(t)ide analogues (NAs) are two classic antiviral drugs (AVDs) for the treatment of chronic hepatitis B (CHB) [14]. And because entecavir (ETV) and tenofovir disoproxil fumarate (TDF) are potent oral NAs that could offer higher antiviral potency and resistance barriers compared to other AVDs, they are currently more widely recommended by both Chinese and international clinical practice guidelines [14–16]. Recently, several studies have shown that patients receiving TDF compared with ETV have a lower risk of HCC occurrence and recurrence [17, 18], while subsequent studies found no significant difference in HCC risk between the two NAs [19–21]. In addition, a growing number of evidence has demonstrated the effectiveness of expanding the indications and applicable population for antiviral therapy for CHB patients [22, 23]. However, whether the early use of AVDs before surgery is beneficial to the prognosis of HCC patients is still unknown, and the expanded indication of antiviral therapy for HCC remains to be further explored.

Therefore, this study aimed to compare the effectiveness of two NAs therapy strategies at different timing on the prognosis of HBV-related HCC patients who received curative resection. And since programmed cell death protein-1 (PD-1) inhibitors are currently considered as one of the most promising treatments for HCC, and their efficacy varies widely among individuals, patients treated with anti-PD-1 therapy were also compared in our study.

## Materials and methods

### Patients and study design

This study retrospectively enrolled 196 HBV-related HCC patients who received radical hepatectomy and either TDF or ETV therapy at Sichuan Provincial People’s Hospital between March 2018 and March 2021. The postoperative adjuvant therapy regimens were formulated according to the China liver cancer (CNLC) staging system [24], American Joint Committee on Cancer 8th edition (AJCC8th), and patient’s wishes. According to the Chinese guidelines for the prevention and treatment of CHB, TDF and ETV were equally recommended as the antiviral therapy during the study period. Moreover, postoperative immunotherapy of all the patients were camrelizumab, which is a PD-1 inhibitor and has been approved by Chinese Food and Drug Administration in HCC [25]. After implementation of the regimen, patients received adjuvant intravenous anti‑PD‑1 therapy every 21 days and continued to receive the treatment until unacceptable toxic reactions were measured or disease progression occurred.

The inclusion criteria were as follows: (1) histopathological confirmation of HCC; (2) Barcelona Clinic Liver Cancer (BCLC) stage 0 to B; (5) pre- or postoperative treatment for CHB with either TDF or ETV; (6) antiviral therapy without change during the entire study period; (7) no preoperative anticancer treatment for primary HCC. The exclusion criteria were as follows: (1) having history of non-HCC malignancies or concurrent with other malignancies; (2) previous antiviral therapy other than ETV or TDF, or combination therapy with NAs; (3) follow-up less than 3 months after hepatectomy or lost to follow-up; (4) initiation of ETV or TDF therapy more than one week after the resection; (5) without systematic screening or detection of co-infections, such as hepatitis C virus, hepatitis D virus, and human immunodeficiency virus; (6) incomplete baseline clinicopathological data. The flowchart of the study design is presented in Supplemental Fig. 1.

This study followed the ethical guidelines of the Helsinki Declaration and was approved by the Human Ethics Committee of Sichuan Academy of Medical Sciences & Sichuan Provincial People’s Hospital (NO.2,023,030). In our center, chief surgeons will inform each HCC patient before surgery that their clinicopathological information could be used for scientific researches (including retrospective studies), ask for their consent, and sign the consent form before the surgery. Therefore, written informed consent was obtained from all participants in the present study.

### Definitions

HBV-related HCC was defined as serum hepatitis B surface antigen (HBsAg) positivity persisting for more than 6 months prior to HCC diagnosis. All preoperative antiviral therapy (ETV or TDF) also initiated for more than 6 months prior to HCC resection. Postoperative ETV or TDF treatment was initiated within one week after resection. Pathological stage was confirmed according to the 7th American Joint Committee on Cancer staging. Cirrhosis was histopathologically determined from the results of the resected liver specimen.

### Follow up

After the surgery, follow-up was preformed every 3 months in the first year and every 6 months thereafter if there was no recurrence or metastasis. During each follow-up, tests of serum alpha-fetoprotein (AFP) levels, liver function, serum HBV DNA levels, as well as abdominal ultrasonography were performed. If signs of recurrence were indicated, CT or MRI was further measured for confirmation. Otherwise, computed tomography (CT) or magnetic resonance imaging (MRI) examinations were conducted every 6 months. The primary outcome was recurrence-free survival (RFS), which was defined as the time from curative resection to the detection of recurrence, metastasis, death, or the end of the follow-up (June 2022). The secondary outcome was overall survival (OS), virological response (VR) and biochemical response (BR). OS was defined as the time from the date of surgery to the date of death, or the end of the follow-up (June 2022). VR was defined as undetectable HBV DNA level (< 1000 copy/mL), and BR was defined as alanine aminotransferase (ALT) < 40 U/L [26].

### Statistical analysis

The associations between antiviral therapy (postoperative only vs. pre- and postoperative) and clinicopathologic characteristics were compared using Pearson’s χ2 test or Fisher’s exact test for categorical variables, and Student’s t test or Mann–Whitney U-test for continuous variables. Continuous data were expressed as mean ± SD. Survival curves for RFS and OS were analyzed by the Kaplan–Meier method and the differences were compared using the log-rank test. Univariate analysis was further performed by the Cox proportional hazards model to identify independent risk factors for HCC recurrence and overall mortality. Multivariate Cox regression analysis was performed for characteristics that were significant in univariate analysis. Nowadays, the Area Under the Curve (AUC) obtained from Receiver Operating Characteristic (ROC) analysis has become an intuitive measure that is widely used in the evaluation of diagnostic tests or predictive models [27, 28]. It could quantify the overall discriminative ability of a test or model across all possible thresholds. Therefore, ROC analysis was also used to evaluate the effect of different risk factors on prognosis. All statistical analyses were carried out using SPSS statistical software (version 23.0; SPSS-IBM, Chicago, IL, USA) and GraphPad Prism (version 9.2.0; GraphPad, CA, USA). Two-tailed P < 0.05 was considered statistically significant.

## Results

### Baseline characteristics

A total of 196 participants were recruited in this study, including 90 (45.9%) patients treated with ENV or TDF only postoperatively, and 106 (54.1%) patients treated with ENV or TDF both preoperatively and postoperatively. The average age was 57 ± 12 years, and 166 (84.7%) patients were male. BCLC stage 0/A and B accounted for 58.7% and 41.3%, respectively. Pathology showed that 76.5% of the tumors were medium-high differentiation. Microvascular invasion (MVI) was noted in 80 (40.8%) patients and 134 (68.4%) patients were confirmed with cirrhosis. After surgery, 44 (22.4%) patients received anti-PD-1 therapy. The median follow-up time of the study was 23.5 (interquartile range (IQR) 16–35) months. The baseline characteristics of all the patients are shown in Table 1.

**Table 1:** Baseline characteristics of patients treated with nucleos(t)ide analogues therapy before and after curative surgery for HBV-related HCC

Characteristics	Postoperative only (n = 90)	Pre- and postoperative (n = 106)	P value
**Gender, male n (%)**	81 (90.0)	85 (80.2)	0.057
**Age, years**	56 ± 12	58 ± 11	0.247
**BMI, kg/m** ^**2**^	22.7 ± 3.0	22.7 ± 3.0	0.806
**HBV DNA, n (%)**			**< 0.001**
< 1000 copy/mL	24 (26.7)	67 (63.2)	
≥ 1000 copy/mL	66 (73.3)	39 (36.8)	
**ALB, g/L**	36.8 ± 5.4	36.5 ± 4.8	0.780
**PT, seconds**	11.9 ± 1.3	12.0 ± 1.5	0.593
**Platelet, 10** ^**9**^ **/L**	155 ± 78	150 ± 84	0.450
**ALT, n (%)**			0.068
< 40 U/L	60 (66.7)	83 (78.3)	
≥ 40 U/L	30 (33.3)	23 (21.7)	
**HBeAg positive, n (%)**	30 (33.3)	31 (29.2)	0.538
**AFP, n (%)**			0.582
< 20 ng/mL	46 (51.1)	50 (47.2)	
≥ 20 ng/mL	44 (48.9)	56 (52.8)	
**Total bilirubin, µmol/L**	22.8 ± 28.7	20.4 ± 17.1	0.502
**BCLC stage**			0.268
0/A	49 (54.4)	66 (62.3)	
B	41 (45.6)	40 (37.7)	
**Microvascular invasion**			**0.007**
Negative	43 (47.8)	69 (65.1)	
Positive	47 (52.2)	37 (34.9)	
**Tumor diameter, cm**	6.4 ± 2.7	6.0 ± 3.5	0.132
**Histopathological type**			0.767
Low	22 (24.4)	24 (22.6)	
Medium-high	68 (75.6)	82 (77.4)	
**Tumor number**			0.328
Single	56 (62.2)	73 (68.9)	
Multiple	34 (37.8)	33 (31.1)	
**Cirrhosis**			0.286
No	25 (27.8)	37 (34.9)	
Yes	65 (72.2)	69 (65.1)	
**Postoperative anti-PD-1 therapy, n (%)**	20 (22.2)	24 (22.6)	0.944

*Note* Bold values mean the P value is significant

*Abbreviations* HBV, hepatitis B virus; HCC, hepatocellular carcinoma; BMI, body mass index; ALB, albumin; PT, prothrombin time; ALT, alanine aminotransferase; HBeAg, hepatitis e antigen; AFP, alpha-fetoprotein; BCLC, Barcelona Clinic Liver Cancer; PD-1, programmed cell death protein 1

Compared with the pre- and postoperative NAs group, the postoperative NAs group had significantly more patients with higher levels of serum HBV DNA (73.3% vs. 36.8%; *P* < 0.001) and more patients with MVI (51.1% vs. 32.1%, *P* = 0.007).

### Effect of NAs therapy at different timing on HCC recurrence

At the end of follow-up, 66 (33.7%) patients presented tumor recurrence, including 44 (48.9%) patients in the postoperative NAs group and 22 (20.8%) patients in the pre- and postoperative NAs group, respectively. The 1-, 2-, and 3-year cumulative recurrence rates for the entire cohort were 25.5% (95% CI: 19.2–31.8%), 32.9% (95% CI: 26.2–39.6%), and 36.6% (95% CI: 29.3–43.9%), respectively.

**Fig. 1:**
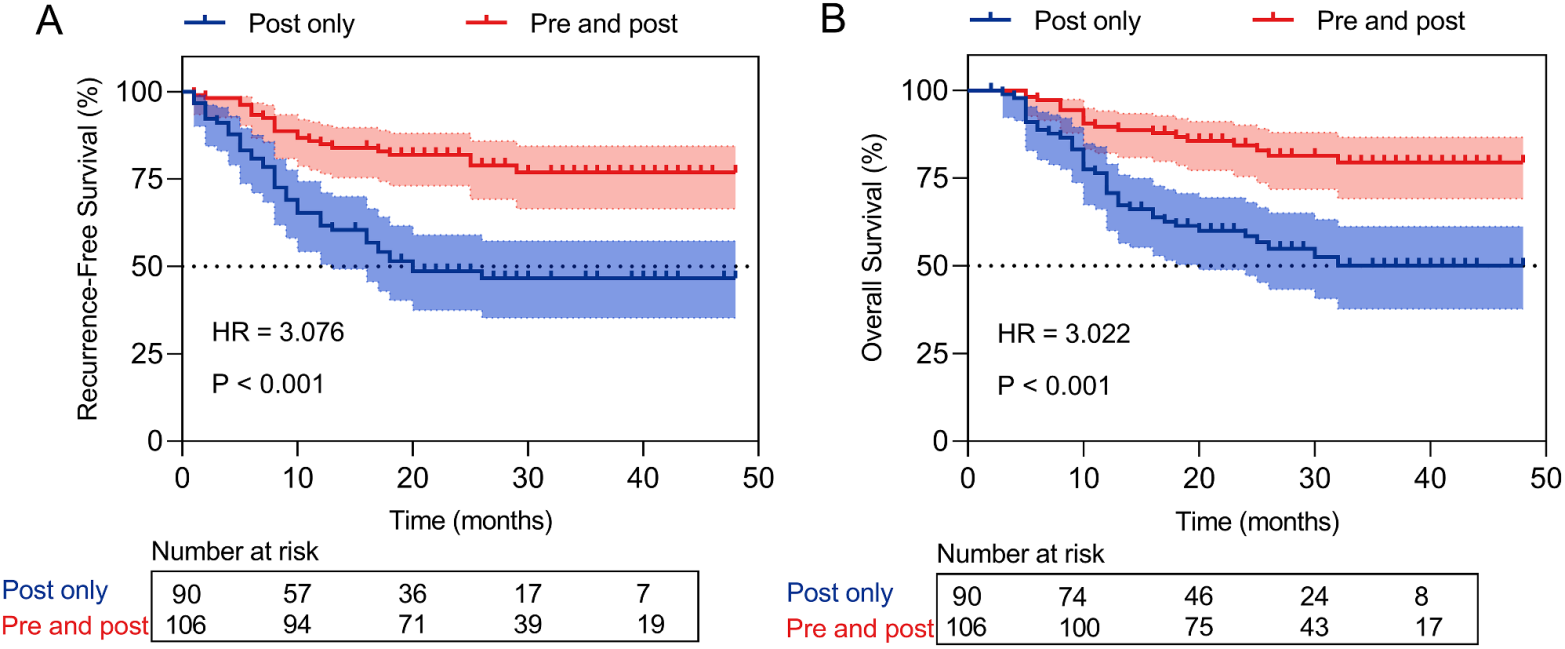
Recurrence-free survival (**A**) and overall survival (**B**) of the patients treated with nucleos(t)ide analogues therapy before and after curative surgery. *Abbreviations* Post, postoperative; pre and post; pre- and postoperative

The pre- and postoperative NAs group had significantly better recurrence-free survival than the postoperative NAs group (HR: 3.076; 95% CI: 1.877–5.039; *P* < 0.001; Fig. 1A). Moreover, the 1-, 2-, and 3-year cumulative recurrence rates for the postoperative NAs group were 38.3% (95% CI: 27.9–48.7%), 51.4% (95% CI: 40.6–62.2%), and 53.4% (95% CI: 42.2–64.6%), respectively. And the 1-, 2-, and 3-year cumulative recurrence rates for the pre- and postoperative NAs group were 15.1% (95% CI: 8.2–22.0%), 18.1% (95% CI: 10.7–25.5%), and 23.1% (95% CI: 14.3–31.9%), respectively. Univariate and multivariate analyses were further performed for clinicopathologic variables. From the multivariate Cox regression analysis, we found that BCLC Stage (HR: 3.842; 95% CI: 1.924–7.671; *P* < 0.001), MVI (HR: 2.421; 95% CI: 1.397–4.197; *P* = 0.002), and NAs therapy (postoperative only vs. pre- and postoperative; HR: 0.468; 95% CI: 0.270–0.814; *P* = 0.007) were significant prognostic factors associated with postoperative recurrence in HBV-related HCC patients (Table 2). The ROC analysis also demonstrated that NAs therapy (postoperative only vs. pre- and postoperative) performed well in predicting postoperative HCC recurrence of the patients (AUC:0.656; 95% CI: 0.585–0.723; Supplemental Table 1).

**Table 2:** Univariate and multivariate Cox regression analyses for recurrence and overall mortality of the HBV-related HCC patients after curative resection

	HCC Recurrence				Overall Mortality			
	Univariate Analysis		Multivariate Analysis		Univariate Analysis		Multivariate Analysis	
Characteristics	HR (95% CI)	P value	HR (95% CI)	P value	HR (95% CI)	P value	HR (95% CI)	P value
Sex (Male vs. Female)	0.491 (0.212–1.136)	0.096			0.469 (0.188–1.173)	0.105		
BCLC Stage (0-A vs. B)	4.204 (2.498–7.076)	**< 0.001**	3.842 (1.924–7.671)	**< 0.001**	5.115 (2.871–9.110)	**< 0.001**	5.020 (2.353–10.708)	**< 0.001**
Tumor number (Single vs. Multiple)	1.865 (1.148–3.030)	**0.012**	0.661 (0.360–1.216)	0.184	1.992 (1.195–3.324)	**0.008**	0.648 (0.342–1.228)	0.183
Microvascular invasion (Negative vs. Positive)	3.526 (2.127–5.843)	**< 0.001**	2.421 (1.397–4.197)	**0.002**	3.199 (1.871–5.468)	**< 0.001**	2.040 (1.136–3.662)	**0.017**
AFP, ng/mL (< 20 vs. ≥20)	1.636 (1.001–2.674)	**0.049**	1.195 (0.713–2.003)	0.499	1.874 (1.105–3.179)	**0.020**	1.349 (0.774–2.349)	0.291
Histopathological type (Low vs. Medium-high)	0.592 (0.347–1.010)	0.054			0.664 (0.378–1.167)	0.154		
Cirrhosis (No vs. Yes)	1.339 (0.778–2.302)	0.292			1.645 (0.902–2.998)	0.104		
HBV DNA (< 1000 copy/mL vs. ≥1000 copy/mL)	0.904 (0.514–1.588)	0.724			0.875 (0.479–1.596)	0.662		
NAs therapy (Group I + II vs. Group III + IV)	0.433 (0.252–0.744)	**0.002**	0.468 (0.270–0.814)	**0.007**	0.410 (0.228–0.737)	**0.003**	0.445 (0.245–0.809)	**0.008**

*Note* Bold values mean the P value is significant

*Abbreviations* HBV, hepatitis B virus; HCC, hepatocellular carcinoma; BCLC, Barcelona Clinic Liver Cancer; AFP, alpha-fetoprotein; NAs, nucleos(t)ide analogues

### Effect of NAs therapy at different timing on overall survival

At the end of follow-up, 59 (30.1%) patients died, including 40 (44.4%) patients in the postoperative NAs group and 19 (17.9%) patients in the pre- and postoperative NAs group, respectively. The 1-, 2-, and 3-year OS rates for the entire cohort were 81.0%, 72.5%, and 66.1%, respectively.

The pre- and postoperative NAs group had significantly better overall survival than the postoperative NAs group (HR: 3.022; 95% CI: 1.799–5.076; *P* < 0.001; Fig. 1B). Moreover, the 1-, 2- and 3-year OS rates were 70.7%, 58.4%, and 50.1% in the postoperative NAs group, and 89.6%, 84.3% and 79.4% in the pre- and postoperative NAs group, respectively (Fig. 1B). Univariate and multivariate analyses were further performed for clinicopathologic variables. From the multivariate Cox regression analysis, we found that BCLC Stage (HR: 5.020; 95% CI: 2.353–10.708; *P* < 0.001), MVI (HR: 2.040; 95% CI: 1.136–3.662; *P* = 0.017), and NAs therapy (postoperative only vs. pre- and postoperative; HR: 0.445; 95% CI: 0.245–0.809; *P* = 0.008) were powerful prognostic factors for postoperative overall mortality in HBV-related HCC patients (Table 2). The ROC analysis also demonstrated that NAs therapy (postoperative only vs. pre- and postoperative) performed well in predicting postoperative overall mortality of the patients (AUC:0.657; 95% CI: 0.585–0.723; Supplemental Table 1).

### Effect of postoperative anti-PD-1 therapy on prognosis of patients with different HBV DNA level

**Fig. 2:**
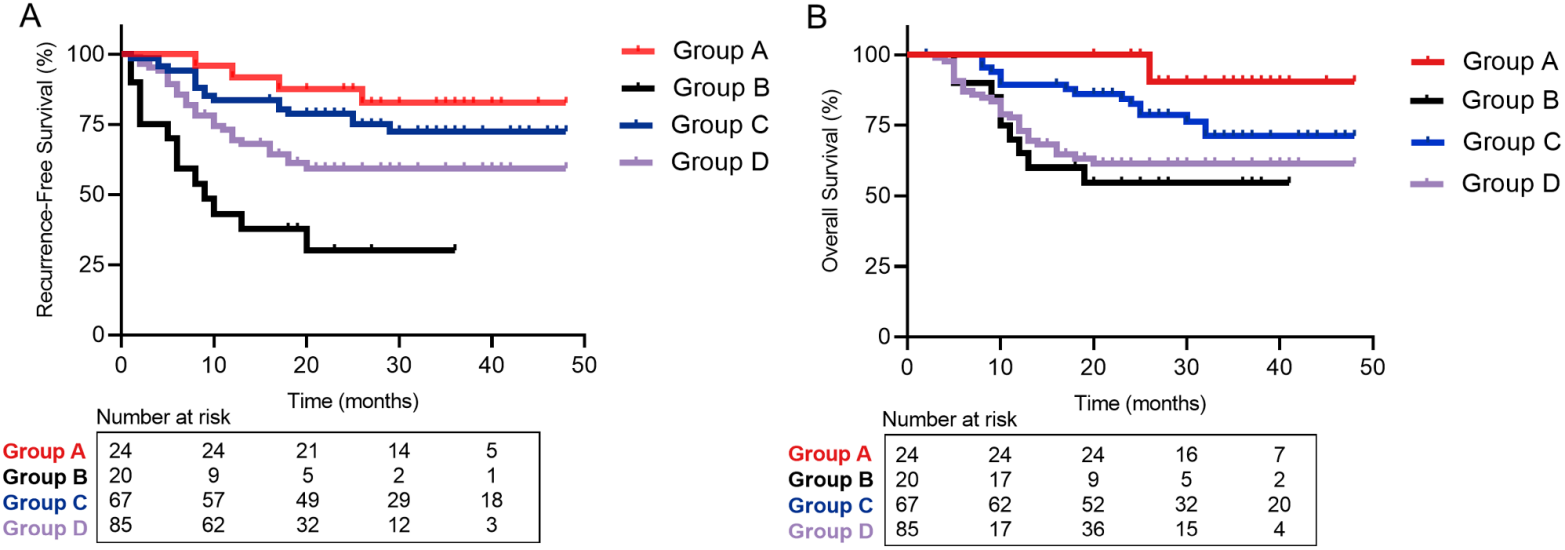
Recurrence-free survival (**A**) and overall survival (**B**) of the patients with or without postoperative anti-PD-1 therapy at different HBV DNA level. *Abbreviations* PD-1, programmed cell death protein 1; HBV, hepatitis B virus. Group A, patients who received anti-PD-1 therapy and HBV DNA < 1000 copy/mL; Group B, patients who received anti-PD-1 therapy and HBV DNA ≥ 1000 copy/mL; Group C, patients without anti-PD-1 therapy and HBV DNA < 1000 copy/mL; Group D, patients without anti-PD-1 therapy and HBV DNA ≥ 1000 copy/mL

According to different HBV DNA level and postoperative anti-PD-1 therapy, the patients were divided into the following four groups: patients who received anti-PD-1 therapy and HBV DNA < 1000 copy/mL were group A; patients who received anti-PD-1 therapy and HBV DNA ≥ 1000 copy/mL were group B; patients without anti-PD-1 therapy and HBV DNA < 1000 copy/mL were group C; patients without anti-PD-1 therapy and HBV DNA ≥ 1000 copy/mL were group D. The relationship between clinicopathological factors and anti-PD-1 therapy are presented in Table 3. Except age, the differences of all other characteristics between patients with and without anti-PD-1 therapy were not statistically significant (*P* > 0.05). Survival curves for RFS and OS of these four groups are presented in Fig. 2.

**Table 3:** Associations of postoperative anti-PD-1 therapy and clinicopathologic characteristics of HBV-related HCC patients with different HBV DNA level

	With anti-PD-1 therapy		Without anti-PD-1 therapy		P value
Characteristics	Group A (n = 24)	Group B (n = 20)	Group C (n = 67)	Group D (n = 85)	
**Gender, male n (%)**	20 (83.3)	18 (90.0)	51 (76.1)	77 (90.6)	0.727
**Age, years**	56 ± 9	50 ± 13	62 ± 11	55 ± 12	**0.015**
**BMI, kg/m** ^**2**^	22.7 ± 3.0	22.7 ± 2.6	22.8 ± 3.1	22.6 ± 3.0	0.710
**ALB, g/L**	36.9 ± 5.8	38.4 ± 3.7	37.1 ± 4.9	35.8 ± 5.2	0.202
**PT, seconds**	12.1 ± 1.4	11.3 ± 0.7	12.0 ± 1.8	12.0 ± 1.2	0.190
**Platelet, 10** ^**9**^ **/L**	140 ± 65	158 ± 71	164 ± 99	145 ± 71	0.947
**ALT, n (%)**					0.320
< 40 U/L	21 (87.5)	9 (45.0)	55 (82.1)	60 (70.6)	
≥ 40 U/L	3 (12.5)	11 (55.0)	12 (17.9)	25 (29.4)	
**HBeAg positive, n (%)**	2 (8.3)	10 (50.0)	5 (7.5)	44 (51.8)	0.856
**AFP, n (%)**					0.595
< 20 ng/mL	12 (50.0)	8 (40.0)	35 (52.2)	41 (48.2)	
≥ 20 ng/mL	12 (50.0)	12 (60.0)	32 (47.8)	44 (51.8)	
**Total bilirubin, µmol/L**	21.3 ± 9.0	17.1 ± 7.5	20.3 ± 20.0	23.6 ± 29.8	0.869
**BCLC stage, n (%)**					0.652
0/A	17 (70.8)	7 (35.0)	47 (70.1)	44 (51.8)	
B	7 (29.2)	13 (65.0)	20 (29.9)	41 (48.2)	
**Tumor diameter, cm**	5.2 ± 2.5	7.9 ± 3.3	6.1 ± 3.1	6.1 ± 3.2	0.324
**Tumor number**					0.179
Single	16 (66.6)	10 (50.0)	51 (76.1)	52 (61.2)	
Multiple	8 (33.3)	10 (50.0)	16 (23.9)	33 (38.8)	
**Cirrhosis**					0.061
No	12 (50.0)	7 (35.0)	25 (37.3)	18 (21.2)	
Yes	12 (50.0)	13 (65.0)	42 (62.7)	67 (78.8)	
**Recurrence**					0.429
No	20 (83.3)	7 (35.0)	50 (74.6)	53 (62.4)	
Yes	4 (16.7)	13 (65.0)	17 (25.4)	32 (37.6)	
**Death**					0.402
No	22 (91.7)	11 (55.0)	51 (76.1)	53 (62.4)	
Yes	2 (8.3)	9 (45.0)	16 (23.9)	32 (37.6)	

*Note* Bold values mean the P value is significant

*Abbreviations*: PD-1, programmed cell death protein 1; HBV, hepatitis B virus; HCC, hepatocellular carcinoma; BMI, body mass index; ALB, albumin; PT, prothrombin time; ALT, alanine aminotransferase; HBeAg, hepatitis e antigen; AFP, alpha-fetoprotein; BCLC, Barcelona Clinic Liver Cancer

When patients received postoperative anti-PD-1 therapy, there were 24 patients with HBV DNA < 1000 copy/mL, and 20 patients with HBV DNA ≥ 1000 copy/mL. Patients in group A were associated with significantly better RFS and OS compared with patients in group B (HR: 7.783; 95% CI: 2.283–26.530; *P* = 0.002; Fig. 2A; HR: 6.699; 95% CI: 2.431–18.46; *P* < 0.001; Fig. 2B). In addition, when patients in BCLC Stage B, patients in group A were also associated with significantly better RFS and OS compared with patients in group B (HR: 5.064; 95% CI: 1.633–15.70; *P* = 0.015; Supplemental Fig. 2A; HR: 6.985; 95% CI: 1.885–25.88; *P* = 0.025; Supplemental Fig. 2B).

Patients in group C and D were associated with significantly better RFS compared with patients in group B (HR: 4.244; 95% CI: 1.569–11.480; *P* < 0.001; Fig. 2A; HR: 2.366; 95% CI: 1.036–5.406; *P* = 0.006; Fig. 2A). However, no difference was observed with OS rates in group B and group D (HR: 1.215; *P* = 0.598; Fig. 2B).

### Early virological and biochemical responses after curative surgery

**Fig. 3:**
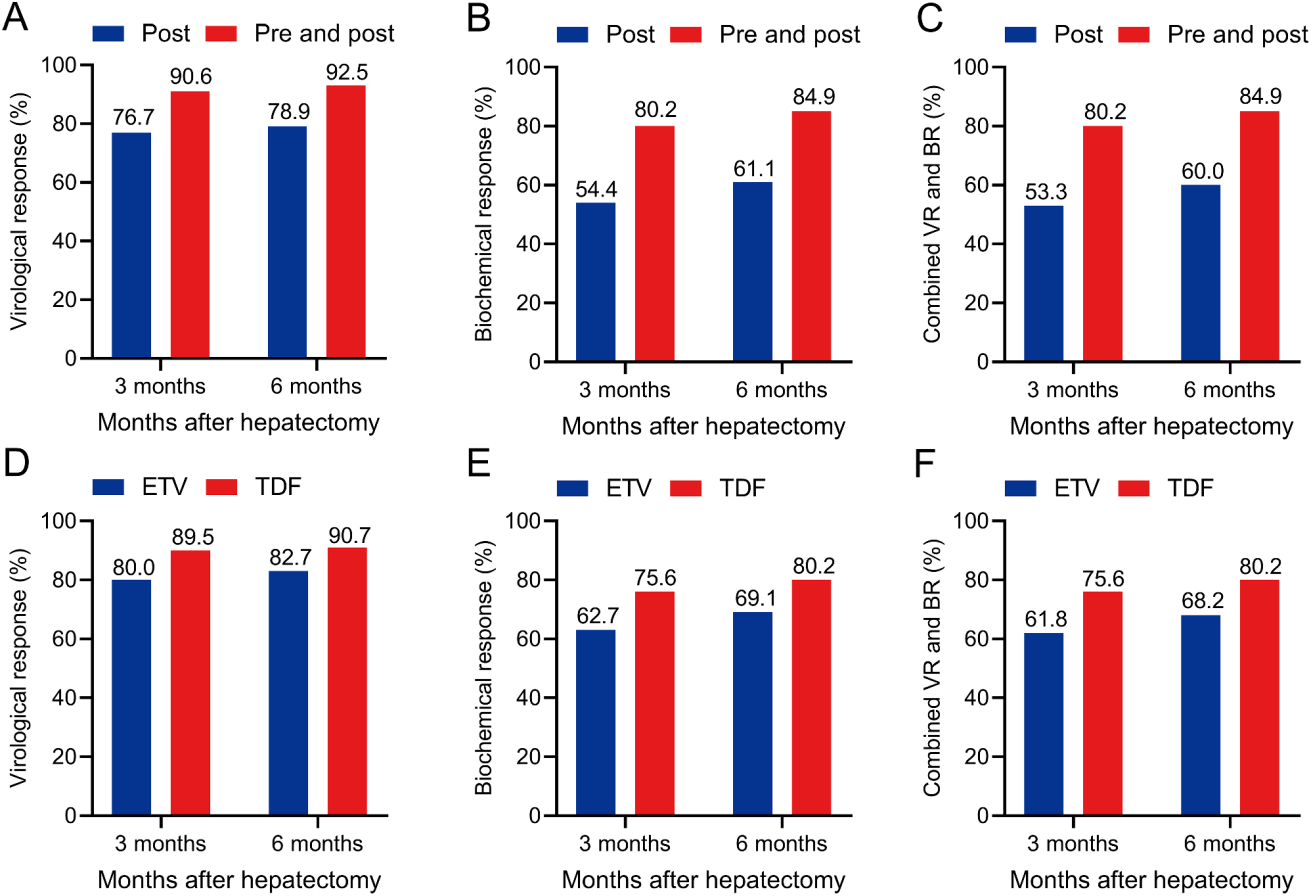
Early virological response, biochemical response and combined virological and biochemical response of the patients treated with nucleos(t)ide analogues therapy before and after curative surgery (**A**, **B**, **C**) and treated with entecavir vs. tenofovir disoproxil fumarate (**D**, **E**, **F**). *Abbreviations* Post, postoperative; pre and post; pre- and postoperative; ETV, entecavir; TDF, tenofovir disoproxil fumarate

The virological and biochemical response of the pre- and postoperative NAs group at the 3rd and 6th months were significantly better than those of the postoperative NAs group (*P* < 0.05; Fig. 3A, B). Similarly, the results were observed in the rates of combined virological and biochemical response at the 3rd and 6th months (80.2% vs. 53.3%; *P* < 0.001; 84.9% vs. 60.0%; *P* < 0.001; Fig. 3C).

The rates of virological and biochemical response at the 3rd and 6th months were similar between the ETV group and the TDF group (VR; 80% vs. 89.5%; *P* = 0.069; 82.7% vs. 90.7%; *P* = 0.108; Fig. 3D; BR; 62.7% vs. 75.6%; *P* = 0.055; 69.1% vs. 80.2%; *P* = 0.078; Fig. 3E). Similar result was observed in the rate of combined virological and biochemical response at the 6th month (68.2% vs. 80.2%; *P* = 0.058; Fig. 3F). However, the combined virological and biochemical response of the TDF group at the 3rd month was significantly better than that of the ETV group (75.6% vs. 61.8%; *P* = 0.041; Fig. 3F).

### Prognostic analysis of ETV and TDF

**Fig. 4:**
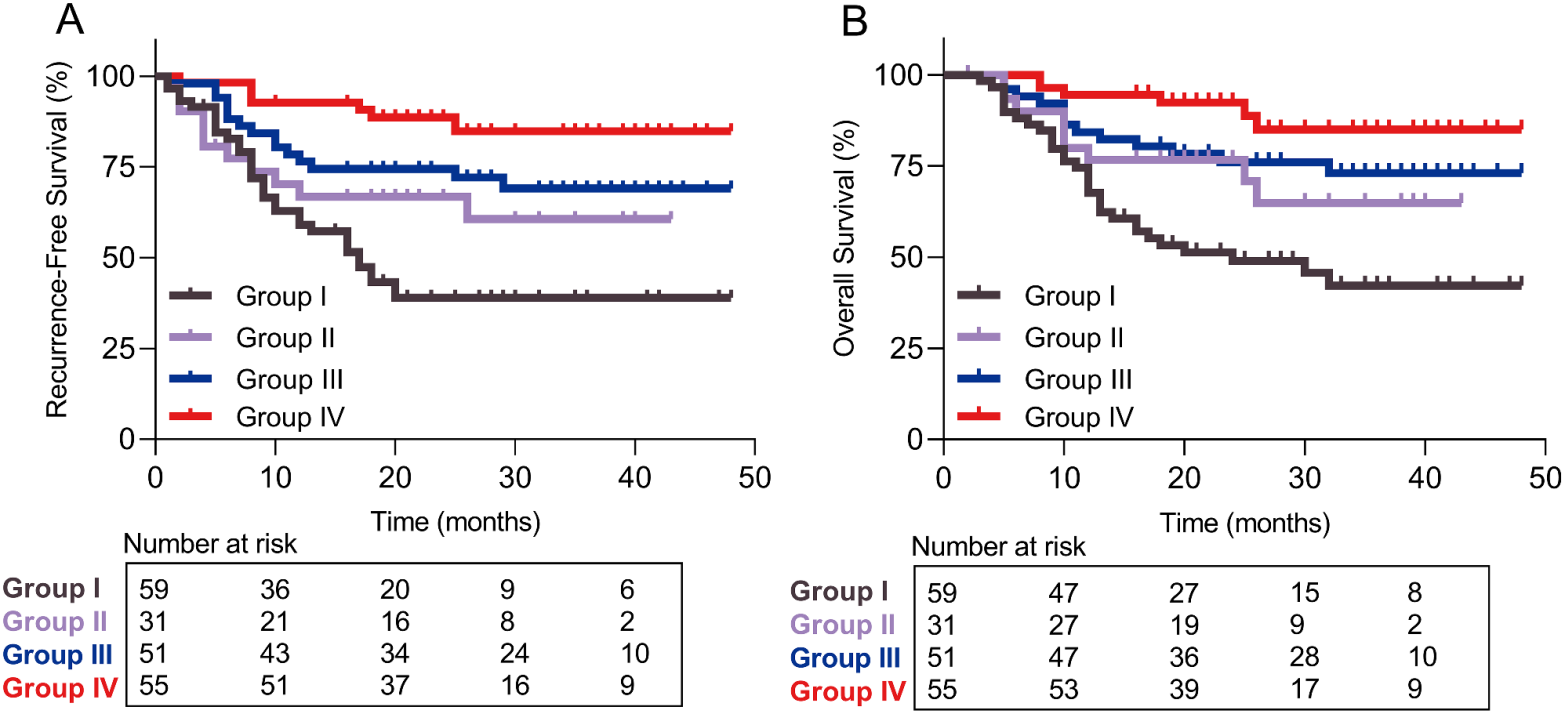
Recurrence-free survival (**A**) and overall survival (**B**) of the patients treated with ETV vs. TDF before and after curative surgery. *Abbreviations* ETV, entecavir; TDF, tenofovir disoproxil fumarate. Group I, patients who received postoperative ETV without preoperative antiviral therapy; Group II, patients who received postoperative TDF without preoperative antiviral therapy; Group III, patients who received pre- and postoperative ETV; Group IV, patients who received pre- and postoperative TDF

In this study, the 196 HBV-related HCC patients were divided into the following four groups: 59 patients who received postoperative ETV without preoperative antiviral therapy were group I; 31 patients who received postoperative TDF without preoperative antiviral therapy were group II; 51 patients who received pre- and postoperative ETV were group III; 55 patients who received pre- and postoperative TDF were group IV (Table 4). When patients treated with TDF, patients in group IV were associated with significantly better RFS and OS compared with patients in group II (HR: 3.381; 95% CI: 1.253–9.123; *P* = 0.007; Fig. 4A; HR: 2.958; 95% CI: 1.014–8.634; *P* = 0.029; Fig. 4B). When patients treated with ETV, patients in group III were associated with significantly better RFS and OS compared with patients in group I (HR: 2.494; 95% CI: 1.415–4.396; *P* = 0.002; Fig. 4A; HR: 2.581; 95% CI: 1.429–4.663; *P* = 0.002; Fig. 4B). No significant difference was observed with RFS and OS rates between group I and group II (HR: 1.701; *P* = 0.115; Fig. 4A; HR: 1.992; *P* = 0.059; Fig. 4B), or between group III and group IV (HR: 2.358; *P* = 0.051; Fig. 4A; HR: 2.245; *P* = 0.089; Fig. 4B).

**Table 4:** Baseline characteristics of patients treated with entecavir vs. tenofovir disoproxil fumarate before and after curative surgery for HBV-related HCC

	Postoperative only		Pre- and postoperative		P value
Characteristics	Group I (n = 59)	Group II (n = 31)	Group III (n = 51)	Group IV (n = 55)	
**Gender, male n (%)**	53 (89.8)	28 (90.3)	40 (78.4)	45 (81.8)	0.057
**Age, years**	56 ± 12	56 ± 14	59 ± 11	57 ± 12	0.247
**BMI, kg/m** ^**2**^	22.9 ± 2.8	22.4 ± 3.2	22.8 ± 3.0	22.8 ± 3.1	0.806
**HBV DNA, n (%)**					**< 0.001**
DNA < 1000 copy/mL	12 (20.3)	12 (38.7)	33 (64.7)	34 (61.8)	
DNA ≥ 1000 copy/mL	47 (79.7)	19 (61.3)	18 (35.3)	21 (38.2)	
**ALB, g/L**	36.2 ± 5.8	37.9 ± 4.4	36.3 ± 5.1	36.7 ± 4.5	0.780
**PT, seconds**	12.1 ± 1.3	11.5 ± 1.0	12.2 ± 1.9	11.9 ± 1.2	0.593
**Platelet, 10** ^**9**^ **/L**	145 ± 82	174 ± 68	166 ± 96	135 ± 70	0.450
**ALT, n (%)**					0.068
< 40 U/L	37 (62.7)	23 (74.2)	36 (70.6)	47 (85.5)	
≥ 40 U/L	22 (37.3)	8 (25.8)	15 (29.4)	8 (14.5)	
**HBeAg positive, n (%)**	14 (23.7)	16 (51.6)	17 (33.3)	14 (25.5)	0.538
**AFP, n (%)**					0.582
< 20 ng/mL	29 (49.2)	17 (54.8)	24 (47.1)	26 (47.3)	
≥ 20 ng/mL	30 (50.8)	14 (45.2)	27 (52.9)	29 (52.7)	
**Total bilirubin, µmol/L**	25.6 ± 34.7	17.4 ± 8.1	21.0 ± 22.6	19.9 ± 9.3	0.502
**BCLC stage**					0.268
0/A	29 (49.2)	20 (64.5)	29 (56.9)	37 (67.3)	
B	30 (50.8)	11 (36.7)	22 (43.1)	18 (32.7)	
**Microvascular invasion**					**0.007**
Negative	29 (49.2)	14 (45.2)	34 (66.7)	35 (63.6)	
Positive	30 (50.8)	17 (54.8)	17 (33.3)	20 (36.4)	
**Tumor diameter, cm**	6.2 ± 2.6	6.7 ± 2.6	6.4 ± 3.8	5.6 ± 3.2	0.132
**Histopathological type**					0.767
Low	12 (20.3)	10 (32.3)	9 (17.6)	15 (27.3)	
Medium-high	47 (79.7)	21 (67.7)	42 (82.4)	40 (72.7)	
**Tumor number**					0.328
Single	34 (57.6)	22 (71.0)	36 (70.6)	37 (67.3)	
Multiple	25 (42.4)	9 (29.0)	15 (29.4)	18 (32.7)	
**Cirrhosis**					0.286
No	14 (23.7)	11 (35.5)	19 (37.3)	18 (32.7)	
Yes	45 (76.3)	20 (64.5)	32 (62.7)	37 (67.3)	
**Postoperative anti-PD-1 therapy, n (%)**	11 (18.6)	9 (29.0)	9 (17.6)	15 (27.3)	0.944

*Note* Bold values mean the P value is significant

*Abbreviations* HBV, hepatitis B virus; HCC, hepatocellular carcinoma; BMI, body mass index; ALB, albumin; PT, prothrombin time; ALT, alanine aminotransferase; HBeAg, hepatitis e antigen; AFP, alpha-fetoprotein; BCLC, Barcelona Clinic Liver Cancer; PD-1, programmed cell death protein 1

## Discussion

In this study, we found that NAs therapy at different timing (postoperative only vs. pre- and postoperative) was a strong independent risk factor for recurrence and overall mortality of HBV-related HCC patients after curative resection. Patients treated with pre- and postoperative NAs (ETV or TDF) therapy had significantly better RFS and OS than those treated with postoperative NAs (ETV or TDF) therapy. Furthermore, in HCC patients received postoperative anti-PD-1 therapy, the results showed that patients with HBV DNA < 1000 copy/mL had significantly better RFS and OS than those with HBV DNA ≥ 1000 copy/mL. Patients without postoperative anti-PD-1 therapy were also associated with significantly better RFS compared with high HBV DNA patients who received anti-PD-1 therapy. However, there was no significant difference in RFS and OS between the ETV group and TDF group. Similar results were also observed in the rates of early VR, BR and combined VR and BR.

The development of HCC after curative resection is affected by a combination of factors, including primary tumor status, viral replication status, inflammatory or immune status, and operation-related factors [29]. For HBV-related HCC patients, the level of viral replication is one of the most significant predictors for tumor recurrence and long-term survival [30, 31]. Several pioneering studies have also shown that regardless of HBV DNA level, as long as the virus is in a replication state, it affects patient prognosis [32, 33]. Thus, the inhibition of HBV viral replication by antiviral therapy is currently widely used to achieve favorable outcomes. In our study, the results showed that MVI was significantly associated with NAs therapy before and after curative surgery. We further found that it was a significant prognostic factor associated with postoperative recurrence and overall mortality in HBV-related HCC patients. This might suggest that the use of NAs (TDF and ETV) therapy have potential efficacy to suppress microvascular invasion. Coincidentally, several interesting studies have showed that HBV infection and active HBV replication are independent risk factors for the development of MVI in HCC patients [34, 35]. Another study also demonstrated that preoperative antiviral treatment was linked to reduced postoperative incidences of MVI for HBV-related HCC [36]. Further research on underlying mechanism and clinical observations are needed to elucidate this finding.

For treatment strategy, biochemical response (representing ALT normalization) and hepatitis e antigen (HBeAg) loss (representing partial immune control) have long been considered by clinical practice guidelines as valuable endpoints [14, 15]. In the present study, patients undergoing curative surgery were divided into two groups based on the use of preoperative NAs therapy, and most of them had normal ALT level and negative HBeAg at the time of surgery. The results showed that NAs therapy at different timing was a strong independent risk factor for HCC recurrence and overall mortality. Moreover, patients treated with pre- and postoperative NAs had significantly better early virological response, biochemical response, and prognosis. Therefore, we believe that even in the case of normal ALT level and negative HBeAg, it is necessarily needed for HBV-related HCC patients to receive timely and reasonable NAs therapy before curative hepatectomy.

Nowadays, immunotherapy targeting immune checkpoints for HCC has developed tremendously. In particular, immune checkpoint blockers targeting PD-1 have been approved for second-line treatment of HCC with sustained clinical responses and prolonged survival [37, 38]. For HBV-related HCC, several studies have shown that an effective immune system could remove antigens (including HBV) invading the host, and the immune response plays a crucial role in the malignant transformation of CHB to HCC [39]. However, chronic HBV infection could deplete immune cells and unbalance the liver immune microenvironment, thus becoming a key factor in accelerating hepatocarcinogenesis [40, 41]. And this may be basis of the synergistic effect between antiviral therapy and anti-PD-1 therapy. The possible mechanism of anti-PD-1 therapy in HBV-related HCC could be explained by the overexpression of PD-1 to inhibit and deplete CD8^+^ resident memory T cells (TRM cells) [41, 42], which have been proved to be enriched in HBV infected liver, and cause a partial immune response [42, 43]. In the present study, low HBV DNA (HBV DNA < 1000 copy/mL) patients who received postoperative anti-PD-1 therapy were associated with significantly better RFS and OS compared with those with high HBV DNA (HBV DNA ≥ 1000 copy/mL). In addition, patients without postoperative anti-PD-1 therapy were also associated with significantly better RFS compared with high HBV DNA patients who received anti-PD-1 therapy. Therefore, we may find the possible synergistic effect between antiviral therapy and anti-PD-1 therapy, and recommend that HBV-related HCC patients who are potential candidates for postoperative anti-PD-1 therapy should receive adequate antiviral therapy before curative surgery to bring HBV DNA to a lower level.

In recent years, NAs therapy has been widely used due to its precise efficacy and safety, and it has also been reported to be associated with reduced recurrence after HCC resection [8, 44–46]. Compared with the low-potency NAs, the high-potency NAs including ETV and TDF had better virological responses and higher resistance barriers [15, 16], thus leading to significantly higher RFS and OS [47]. Practice guidelines have equally recommended them as first-line AVDs for CHB patients [14–16]. However, although several recent studies have shown that patients treated with TDF had significantly lower rate of postoperative recurrence or mortality than patients treated with ETV [18, 26, 48], it is still controversial whether the two high-potency antiviral drugs (ETV and TDF) differ in the prognosis of patients with HBV-related HCC after curative resection [19, 20]. In our study, although the differences of RFS and OS rates between ETV group and TDF group were not statistically significant (*P* > 0.05), we found that the combined virological and biochemical response of the TDF group at the 3rd month was significantly better than that of the ETV group (75.6% vs. 61.8%; *P* = 0.041; Fig. 3F), indicating that the different response of these two NAs groups might be mainly caused by the difference of antiviral effect. In recent studies, Murata et al. found that patients treated with TDF had higher levels of serum interferon-λ3 [49], and peripheral blood mononuclear cells pretreated with TDF could inhibit the production of interleukin (IL)-10 and induce IL-12p70 and tumor necrosis factor-α [50]. However, these results were not shown in the ETV group. Studies have also shown that interferon-k3 participates in immune regulation during viral infection or autoimmune disorders [51], while IL-10 and IL-12 are closely related to the functions of T cells and NK cells [18]. Although the differences of RFS and OS rates between ETV and TDF group were not statistically significant in the present study, further research on underlying mechanism and clinical observations are needed to elucidate the potential favorable outcomes of TDF.

This study also has several limitations. First, since our study is retrospective, the results may be affected by sample size and selection bias. Therefore, we strictly controlled the inclusion and exclusion criteria, and use objective indicators of the data as much as possible. Second, the patients of the study only come from one center, and the sample size is limited. Further large-scale multicenter studies and randomized controlled trials are needed to validate our findings.

## Conclusion

Timely and reasonable preoperative NAs therapy is necessarily needed to improve the prognosis of patients with HBV-related HCC, even in the case of normal ALT level and negative HBeAg. Furthermore, because the underlying synergistic effect between antiviral therapy and anti-PD-1 therapy is vital to improve the prognosis of patients with HBV-related HCC, further studies are needed to validate our findings.

### Electronic supplementary material

Below is the link to the electronic supplementary material.

Supplementary Material 1

Supplementary Material 2

Supplementary Material 3

## Data Availability

The data that support the findings of this study are available from the corresponding author upon reasonable request.
