# Correction: Impact of preoperative antiviral therapy on the prognosis of hepatitis B virus-related hepatocellular carcinoma

**DOI:** 10.1186/s12885-024-12108-w

**Published:** 2024-03-15

**Authors:** Yuxin Liang, Deyuan Zhong, Zilong Zhang, Yuhao Su, Su Yan, Chunyou Lai, Yutong Yao, Ying Shi, Xiaolun Huang, Jin Shang

**Affiliations:** 1 Present Address: Liver Transplantation Center and HBP Surgery, Sichuan Cancer Hospital & Institute, Sichuan Cancer Center, School of Medicine, University of Electronic Science and Technology of China, Chengdu, China; 2 Department of Hepatobiliary-Pancreatic Surgery, Cell Transplantation Center, Sichuan Provincial People’s Hospital, University of Electronic Science and Technology of China, No. 32, West Second Section, First Ring Road, Qingyang District, Chengdu, 610072 China; 3 Clinical Immunology Translational Medicine Key Laboratory of Sichuan Province & Organ Transplant Research Institute, Sichuan Provincial People’s Hospital, University of Electronic Science and Technology of China, No. 32, West Second Section, First Ring Road, Qingyang District, Chengdu, 610072 China; 4 Department of Hepatobiliary-Pancreatic and Hernia Surgery, Puai Hospital, Tongji Medical College, Wuhan Fourth Hospital, Huazhong University of Science and Technology, Wuhan, 430033 China

**Correction: BMC Cancer 24, 291 (2024)**

10.1186/s12885-024-12031-0

Following publication of the original article [1], the author noticed a typesetting error. The equal contributions statement was erroneously omitted.

The equal contributions statement is as follows: Yuxin Liang and Deyuan Zhong contributed equally to this manuscript.

Further to this, affiliation 1 has been corrected. The correct details are: 1. Liver Transplantation Center and HBP Surgery, Sichuan Cancer Hospital & Institute, Sichuan Cancer Center, School of Medicine, University of Electronic Science and Technology of China, Chengdu, China.

The original article [1] has been corrected.
