# Supplementary material for: Impact of preoperative antiviral therapy on the prognosis of hepatitis B virus-related hepatocellular carcinoma
Source: BMC Cancer. 2024 Mar 4;24:291. doi: 10.1186/s12885-024-12031-0 (PMC10913258; doi:10.1186/s12885-024-12031-0)
Supplement: Supplementary file 1 — Supplementary Material 1 [file 12885_2024_12031_MOESM1_ESM.docx]

**Supplemental Figure 2** Recurrence-free survival (A) and overall survival (B) of the patients with different HBV DNA level in BCLC Stage B after postoperative anti-PD-1 therapy.


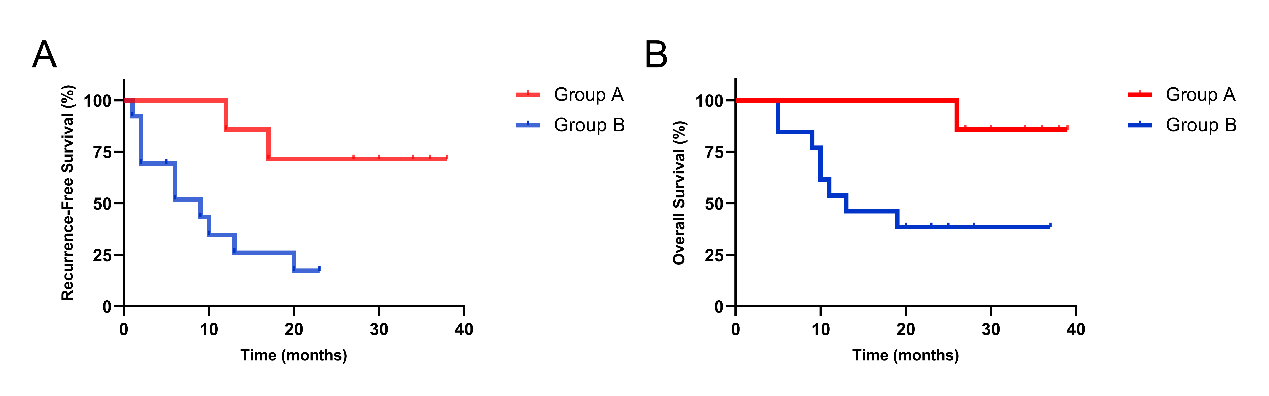
**Abbreviations**: HBV, hepatitis B virus; BCLC, Barcelona Clinic Liver Cancer; PD-1, programmed cell death protein 1. Group A, patients who received anti-PD-1 therapy and HBV DNA <1000 copy/mL; Group B, patients who received anti-PD-1 therapy and HBV DNA ≥1000 copy/mL.
