# Supplementary material for: Impact of preoperative antiviral therapy on the prognosis of hepatitis B virus-related hepatocellular carcinoma
Source: BMC Cancer. 2024 Mar 4;24:291. doi: 10.1186/s12885-024-12031-0 (PMC10913258; doi:10.1186/s12885-024-12031-0)
Supplement: Supplementary file 2 — Supplementary Material 2 [file 12885_2024_12031_MOESM2_ESM.docx]

**Supplemental Table 1** ROC analysis between NAs therapy at different timing and other indicators in recurrence and overall mortality of the HBV-related HCC patients.

|  | **HCC Recurrence** | | **Overall** **Mortality** | |
| --- | --- | --- | --- | --- |
| **Variables** | **AUC (95% CI)** | **P value** | **AUC (95% CI)** | **P value** |
| BCLC stage | 0.702 (0.624-0.781) | **<0.001** | 0.726 (0.647-0.804) | **<0.001** |
| Tumor diameter | 0.734 (0.658-0.810) | **<0.001** | 0.787 (0.717-0.856) | **<0.001** |
| Tumor number | 0.585 (0.499-0.671) | 0.052 | 0.595 (0.507-0.683) | **0.035** |
| Microvascular invasion | 0.672 (0.591-0.753) | **<0.001** | 0.669 (0.585-0.753) | **<0.001** |
| HBV DNA | 0.610 (0.527-0.693) | **0.012** | 0.614 (0.529-0.699) | **0.012** |
| NAs therapy  (Postoperative only vs. pre- and postoperative) | 0.656 (0.585-0.723) | **<0.001** | 0.657 (0.585-0.723) | **<0.001** |

**Abbreviations**: ROC, receiver operating characteristics; NAs therapy, nucleos(t)ide analogues therapy; HBV, hepatitis B virus; HCC, hepatocellular carcinoma; BCLC, Barcelona Clinic Liver Cancer.

**Note**: Bold values mean the P value is significant.
