# Supplementary material for: Impact of preoperative antiviral therapy on the prognosis of hepatitis B virus-related hepatocellular carcinoma
Source: BMC Cancer. 2024 Mar 4;24:291. doi: 10.1186/s12885-024-12031-0 (PMC10913258; doi:10.1186/s12885-024-12031-0)
Supplement: Supplementary file 3 — Supplementary Material 3 [file 12885_2024_12031_MOESM3_ESM.docx]

**Supplemental Figure 1** The flowchart of patients enrolled in this study.


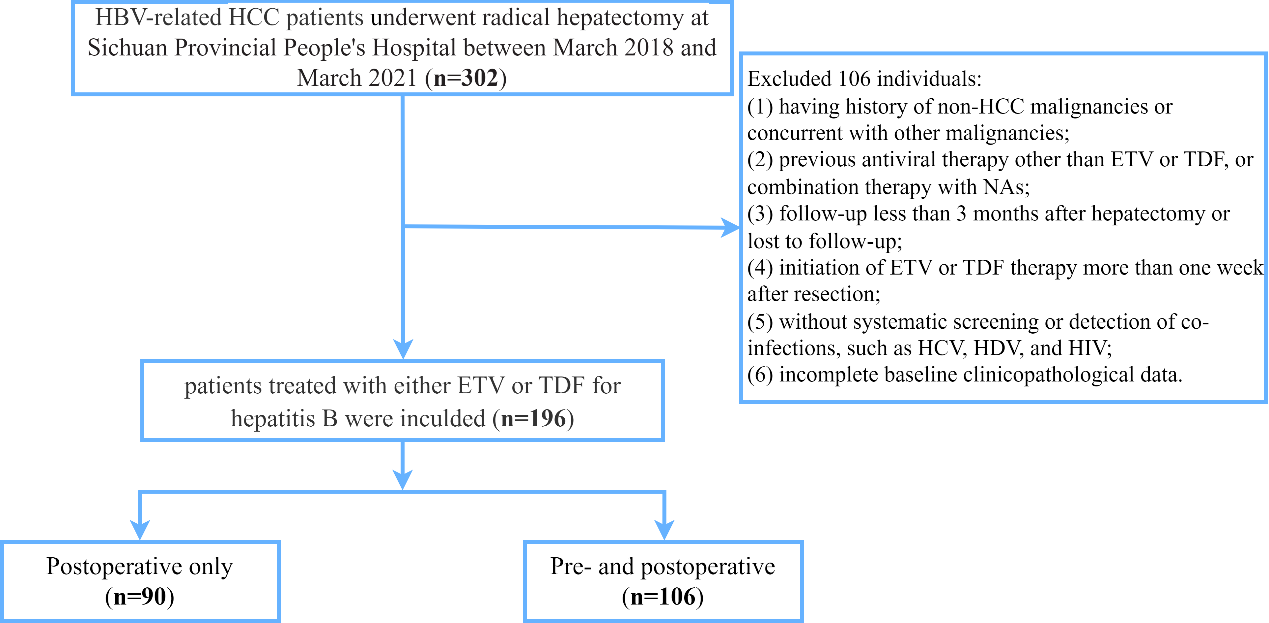


**Abbreviations**: HBV, hepatitis B virus; HCC, hepatocellular carcinoma; ETV, entecavir; TDF, tenofovir disoproxil fumarate; NAs, nucleos(t)ide analogues, HCV, hepatitis C virus; HDV, hepatitis D virus; HIV, human immunodeficiency virus.
